# 碳纳米管复合材料结合分散固相萃取-高效液相色谱-串联质谱法检测环境水样中痕量全氟化合物

**DOI:** 10.3724/SP.J.1123.2022.09016

**Published:** 2023-05-08

**Authors:** Xinli SONG, Ning WANG, Feiyan HE, Canling CHENG, Fei WANG, Jinglong WANG, Lihua ZHANG

**Affiliations:** 枣庄学院食品科学与制药工程学院,山东枣庄277160; College of Food Science and Pharmaceutical Engineering, Zaozhuang University, Zaozhuang 277160, China

**Keywords:** 碳纳米管复合材料, 分散固相萃取, 液相色谱-串联质谱, 全氟化合物, 环境水样, carbon nanotube composite materials, dispersive solid-phase extraction (d-SPE), liquid chromatography-tandem mass spectrometry (LC-MS/MS), perfluorinated compounds (PFCs), environmental water sample

## Abstract

以碳纳米管-二氧化硅棒复合材料为吸附剂,基于分散固相萃取法和液相色谱-串联质谱法建立了一种高灵敏、快速分析环境水样中痕量全氟化合物(PFCs)的方法。该研究选择全氟己烷磺酸、全氟庚酸、全氟辛酸、全氟辛烷磺酸、全氟壬酸和全氟癸酸6种全氟化合物为目标分析物。采用单因素优化法对影响萃取效果的重要因素进行了优化。获得的最佳条件为:吸附时间为30 min、吸附剂用量为10 mg、样品溶液pH为6、萃取过程添加NaCl浓度为1.7 mol/L、解吸溶剂为丙酮、解吸时间为4 min、解吸液的体积为4 mL。采用高效液相色谱-三重四极杆质谱联用技术对水样品中全氟化合物进行定量分析。以5 mmol/L乙酸铵和甲醇为流动相进行梯度洗脱,经Kinetex C18色谱柱(100 mm×2.1 mm, 1.7 μm)分离,采用电喷雾离子源、负离子扫描模式和质谱多反应监测,实现了环境水样中6种全氟化合物的快速定性和定量分析。在优化条件下,6种全氟化合物在各自的线性范围内线性关系良好,检出限(*S/N*=3)为0.10~0.26 ng/L。添加500 ng/L 6种PFCs进行重复性实验,日内相对标准偏差(RSD)为2.51%~7.48%,日间RSD为3.59%~9.63%。将方法应用于自来水、桶装饮用水和河水3种实际环境水样中全氟化合物的分析,在低、中、高3个水平下,6种全氟化合物的加标回收率为72.1%~109.6%,结果满意。本方法成功地应用于实际环境水样中全氟化合物的检测,为快速、有效地检测环境水样中痕量全氟化合物提供了良好的选择。

## Abstract (en)

In this work, carbon nanotubes (CNTs) on silica rod (SiO_2_) composite materials were prepared to extract six perfluorinated compounds (PFCs) in real environmental water samples by high performance liquid chromatography-tandem mass spectrometry (HPLC-MS/MS). The as-synthesized sorbents, hereafter referred to as CNT@SiO_2_, were employed for dispersive solid-phase extraction (d-SPE). Perfluoroheptanoic acid (PFHpA), perfluorohexane sulfonate (PFHxS), perfluorooctanoic acid (PFOA), perfluorononanoic acid (PFNA), perfluorooctane sulfonate (PFOS), and perfluorodecanoic acid (PFDA) were selected as target analytes. The main extraction parameters were systematically optimized using the single-factor optimization method. The optimum adsorption parameters were as follows: adsorption time of 30 min, sorbent amount of 10 mg, pH 6 and NaCl concentration of 1.7 mol/L for sample solution, and 4 mL acetone as desorption solvent, desorption for 4 min. LC-triple quadrupole MS was conducted to quantify the selected PFCs in water samples. The mobile phase was 5 mmol/L ammonium acetate and methanol, the flow rate was set to 0.4 mL/min, the column temperature was set to 40 ℃, and the injection volume was 5.0 μL. The chromatographic separation system was equipped with a Kinetex C18 column (100 mm×2.1 mm, 1.7 μm). The mass spectrometer was operated with negative electrospray ionization in multi-reaction monitoring mode. CNT@SiO_2_ was prepared in five batches and used as the d-SPE sorbent, and the relative standard deviations (RSDs) of the PFC recoveries among these five batches ranged from 4.9% to 9.3%. The reusability of the CNT@SiO_2_ sorbent was assessed. After eight d-SPE cycles using the same sorbent, the RSDs of the PFC recoveries were 3.7%-8.2%. These results indicated that the sorbent had good stability and reusability for d-SPE. Excellent results were achieved under optimal extraction conditions. The method validation results indicated that the linear ranges were 0.4-1000 ng/L for PFNA, PFOS, and PFDA, 0.9-1000 ng/L for PFHpA, 0.7-1000 ng/L for PFHxS, and 0.6-1000 ng/L for PFOA. The correlation coefficients were 0.973-0.997. The limit of detection (LOD) and limit of quantification of the method were 0.10-0.26 ng/L and 0.33-0.87 ng/L, respectively. At 20 ng/L, the RSDs of the intra- and inter-day precisions were 2.73%-7.75% and 3.38%-8.21%, respectively. At 100 ng/L, the RSDs of the intra- and inter-day precisions were 2.95%-8.46% and 4.16%-9.14%, respectively. Finally, at 500 ng/L, the RSDs of the intra- and inter-day precisions were 2.51%-7.48% and 3.59%-9.63%, respectively. The developed method was applied to analyze six PFCs in tap water, barreled drinking water, and river water samples. PFOA and PFOS were determined in tap water at mass concentrations of 5.6 and 8.7 ng/L, respectively. No PFCs were found in barreled drinking water and river water. Satisfactory recoveries of 72.1%-109.6% at low, middle, and high spiking levels were also obtained. In conclusion, the d-SPE-LC-MS/MS method based on CNT@SiO_2_ composite sorbents is accurate and sensitive. The results of this study demonstrate that CNT@SiO_2_ is a good choice for the rapid and effective determination of PFCs from water samples. Further exploration of the use of CNT@SiO_2_ sorbents for the extraction and determination of trace organic pollutions in environmental samples is in progress.

全氟化合物(PFCs)是一类持久性有机污染物,具有较高的表面活性和化学稳定性。PFCs具有持久性和生物累积性,在生物体内毒性蓄积水平高。其次,PFCs还具有生殖毒性、诱变毒性、发育毒性等多种毒性,是一类具有人体内多种脏器毒性的环境污染物^[1,2]^。PFCs主要分布在水、生物样品、土壤和沉积物等物质中,最终进入环境水体^[3⇓-5]^。PFCs的分布十分广泛,易对人类健康和环境造成潜在威胁。因此,当前迫切需要建立一种快速、简单、灵敏的分析方法来检测环境水样中的PFCs。

与气相色谱法测定PFCs相比,液相色谱法不需要衍生化操作,检测灵敏度高,近年来常用于PFCs新型污染物的检测。液相色谱-串联质谱(LC-MS/MS)的多反应监测(MRM)模式,选择性好,干扰少,检出限低,在检测分析中具有显著优势。由于PFCs在环境水样中的痕量分布以及基体效应的干扰,在定量分析检测时,样品前处理是必不可少的环节。样品前处理技术种类繁多,其中,分散固相萃取(d-SPE)技术是在固相萃取(SPE)的基础上发展起来的一种新型固相萃取技术^[6,7]^。它的萃取效率较传统SPE更高,因为在d-SPE过程中,吸附剂在样品溶液中分散均匀,与目标化合物接触更完全。传统的液液萃取、液固萃取、消解萃取等前处理方法操作步骤多、萃取时间长、重现性较差。另外,d-SPE技术所用的有机溶剂使用量少,成本较低,避免了传统SPE技术中上样时间长、高压、易堵塞等缺点^[8,9]^。吸附剂在d-SPE技术中发挥重要作用。目前,已有石墨相氮化碳(g-C_3_N_4_)^[1]^、石墨化炭黑^[10]^等多种新型材料作为d-SPE的吸附剂,并成功应用于水体和食用油中PFCs的分析。

碳纳米管(CNT)是一种富电子、疏水的纳米材料,具有高表面积和强吸附能力,是一种非常优良的吸附剂^[11,12]^。由于CNT比较容易团聚,对于吸附效果有一定影响,因此在CNT中引入了二氧化硅棒作为模板。CNT在二氧化硅棒的表面均匀覆盖,形成核壳立体结构,增加了CNT和目标分析物的接触面积,提高了吸附剂材料对目标分析物的吸附效果。二氧化硅棒模板合成简单,重复性好,易于操作^[13]^。本研究将CNT@SiO_2_作为d-SPE的吸附剂,采用LC-MS/MS法对水样中最常见的6种PFCs(全氟己烷磺酸(PFHxS)、全氟庚酸(PFHpA)、全氟辛酸(PFOA)、全氟辛烷磺酸(PFOS)、全氟壬酸(PFNA)和全氟癸酸(PFDA))进行测定。采用单因素实验设计对影响萃取效率的重要参数进行优化,建立了实际水样中痕量PFCs的检测方法。该方法操作步骤简单,检测灵敏度高,定量准确可靠,可满足环境水样中多种常见PFCs的痕量检测要求。

## 1 实验部分

### 1.1 仪器、试剂与材料

液相色谱-三重四极杆质谱仪(SCIEX Qtrap5500+,美国AB SCIEX公司)。扫描电子显微镜(ZEISS SUPPA^TM^ 55,德国蔡司公司)。傅里叶变换红外光谱仪(FT-IR650,天津港东科技有限公司)。

柠檬酸钠(纯度≥99%)、浓氨水(28%~30%)、聚乙烯吡咯烷酮(*M*_r_ 40000)和正戊醇(纯度≥99%)购自美国Sigma-Aldrich公司。硅酸四乙酯(纯度98%)、聚(4-苯乙烯磺酸钠)(PSS, *M*_r_ 70000)和聚(二烯丙基二甲基氯化铵)(PDDA, *M*_r_ 400000)购自阿拉丁试剂有限公司。二茂铁(纯度98%)购自天津光复精细化工研究所。吐温80购自上海青析化工科技有限公司。过氧化氢(H_2_O_2_,含量30%)购自天津风船化学试剂科技有限公司。羧基化多壁碳纳米管购自南京先丰纳米材料科技有限公司。PFHxS、PFHpA、PFOA、PFOS、PFNA和PFDA标准溶液(质量浓度均为10 mg/mL)购自上海安谱实验科技股份有限公司。用甲醇稀释6种全氟化合物标准溶液,配制成1 μg/mL的标准混合溶液,并在4 ℃下避光保存。使用时稀释至所需浓度。桶装水购于山东国新力源水业有限公司,自来水和河水从学校(枣庄)收集。所有水样均过0.22 μm的水相微孔滤膜,过滤后在棕色玻璃瓶中于4 ℃保存。

### 1.2 CNT@SiO_2_的制备

#### 1.2.1 SiO_2_微米棒的制备^[14]^

将1 g聚乙烯吡咯烷酮溶于10 mL正戊醇中,超声2 h,加入280 μL水、100 μL柠檬酸钠(180 mmol/L)、200 μL浓氨水和1 mL乙醇并振荡1 min;再加入100 μL硅酸四乙酯,振荡1 min后静置,水解20 h。用无水乙醇洗涤3次,真空干燥12 h,可得白色粉末。

#### 1.2.2 CNT@SiO_2_的制备

称30 mg SiO_2_微米棒,加入到50 mL PDDA水溶液(2 mg/mL)中,室温搅拌2 h,离心分离和水洗3次,完成一次PDDA吸附的操作;然后将吸附有PDDA的SiO_2_加入到50 mL的PSS水溶液(2 mg/mL)中室温搅拌2 h,用去离子水洗涤3次后,完成一次PSS吸附到SiO_2_表面的操作;随后再重复进行一次PDDA的吸附操作,在SiO_2_微米棒表面形成了PDDA/PSS/PDDA的3层电解质,此时,SiO_2_微米棒的表面均匀地带有正电荷^[15]^。将以上吸附有聚电解质的SiO_2_微米棒加入一定量的去离子水,配成均匀的悬浮液,室温搅拌,滴加CNT的水溶液(0.5 mg/mL) 20 mL,继续搅拌0.5 h。然后离心分离和水洗3次,完成一次CNT吸附到SiO_2_表面的操作。接着交替吸附PDDA和CNT,重复5次上述操作,可得SiO_2_微米棒表面组装多层碳纳米管的复合材料。

### 1.3 d-SPE过程

将含有1.7 mol/L NaCl的10 mL环境水样(溶液pH=6)置于50 mL离心管中,称10 mg CNT@SiO_2_材料作为吸附剂加入水样中,振荡萃取30 min。萃取完成后离心分离,弃去上清液。下层沉淀物中加入4 mL丙酮解吸液,充分振荡4 min后,离心收集解吸液,将所得解吸液在30 ℃下氮气流吹干。再用100 μL的甲醇复溶并转移至进样瓶中,取5 μL进行LC-MS/MS分析。

### 1.4 液相色谱-质谱条件

色谱条件:色谱柱Kinetex C18柱(100 mm×2.1 mm, 1.7 μm,美国菲罗门公司);柱温为40 ℃,流动相使用5 mmol/L乙酸铵(A)和甲醇(B)^[16]^,流速为0.4 mL/min,进样量为5.0 μL。梯度洗脱:0~1 min, 10%B; 1~1.5 min, 10%B~40%B; 1.5~12 min, 40%B~95%B; 12~13 min, 95%B; 13~17 min, 95%B~10%B。

质谱条件:质谱仪设置为电喷雾负离子模式,采用MRM模式。离子源温度为550 ℃。喷雾电压为-4500 V,雾化气和碰撞气体均为N_2_。雾化气压力为414 kPa (60 psi)。其他参数见表1。

**表1:** 6种PFCs的质谱参数

Compound	Retention time/min	Quantitive ion pair			Qualitative ion pair		
		Collision energy/eV	Monitored transition (*m/z*)		Collision energy/eV		Monitored transition (*m/z*)
Perfluoroheptanoic acid (PFHpA)	5.7	-14	362.9/319.1		-24		362.9/169.0
Perfluorohexane sulfonate (PFHxS)	5.8	-14	399.0/79.8		-24		399.0/99.0
Perfluorooctanoic acid (PFOA)	6.7	-16	413.1/368.9		-24		413.1/169.1
Perfluorononanoic acid (PFNA)	7.6	-18	462.9/418.9		-26		462.9/219.1
Perfluorooctane sulfonate (PFOS)	7.6	-52	499.0/79.9		-57		499.0/99.0
Perfluorodecanoic acid (PFDA)	8.3	-62	512.9/468.9		-67		512.9/218.7

## 2 结果与讨论

### 2.1 CNT@SiO_2_的表征

CNT@SiO_2_吸附材料的扫描电镜如图1所示。图1a显示的是组装1层PDDA/CNT的二氧化硅棒。二氧化硅棒的长度约为3 μm,羧基化多壁碳纳米管长度为0.5~2 μm,直径为20~30 nm,羧酸质量分数为1.23%。CNT围绕在SiO_2_棒的周围,因为CNT的强吸附性和柔韧性保证了它自身能够经过层层组装后,可以沉积分布在SiO_2_棒表面上。组装1层PDDA/CNT后,CNT能够部分覆盖在聚电解质PDDA修饰的SiO_2_棒的表面,但是CNT排列很随机,SiO_2_棒存在部分裸露的部位,组装了5层PDDA/CNT后,CNT已经均匀地分布在聚电解质PDDA修饰的SiO_2_棒表面,形成了完全覆盖、相对致密、厚度可控的CNT层(图1b)。5层组装后,羧酸化碳纳米管在SiO_2_棒上的负载量为19%。

**图1:**
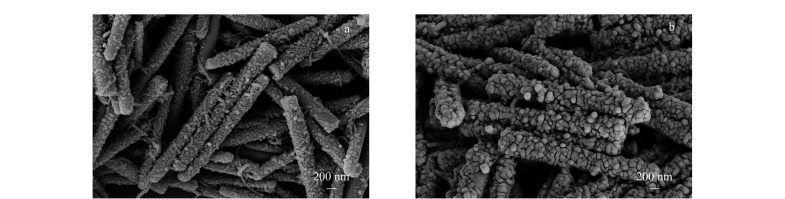
CNT组装在SiO_2_棒上(a)1次和(b)5次的扫描电子显微镜图

### 2.2 CNT@SiO_2_的稳定性

实验中将CNT@SiO_2_作为d-SPE的吸附剂,需要对其化学稳定性进行考察。CNT@SiO_2_在空气中放置72 h与在pH 2的酸性水溶液、pH 10的碱性水溶液、中性丙酮中分别浸泡72 h,经FT-IR分析显示没有明显的差异(图2),这说明此材料在上述条件下均具有良好的稳定性。因此,CNT@SiO_2_可作为d-SPE吸附剂。

**图2:**
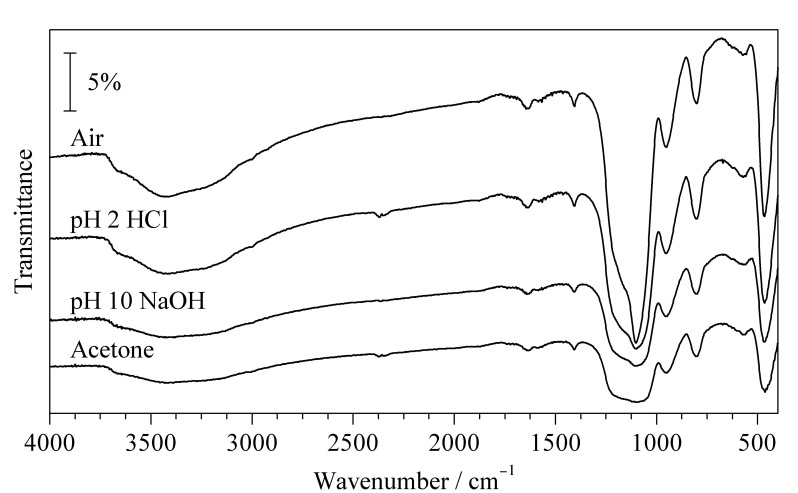
CNT@SiO_2_材料在空气、pH 2酸性条件、pH 10碱性条件和丙酮中的红外光谱图

### 2.3 实验参数的优化

为了评价CNT@SiO_2_作为d-SPE吸附剂的可行性,选择PFHxS、PFHpA、PFOA、PFOS、PFNA和PFDA 6种PFCs(500 ng/L)为目标化合物,以这些化合物的回收率评价CNT@SiO_2_材料对PFCs的萃取效果。

#### 2.3.1 吸附过程参数的优化

本文将d-SPE过程分为吸附和解吸两个步骤,通过单因素实验对吸附步骤进行优化。在吸附步骤中,考察了吸附时间、吸附剂用量、样品pH值和离子强度4个因素。

对吸附时间在5~60 min内的萃取效果进行了考察。如图3a所示,在5~30 min范围内,回收率随着时间的增加而增大;在30~60 min范围内,回收率基本不变。富集30 min后回收率趋于平缓,表明已接近饱和状态,此时目标物在吸附剂和溶液之间达到了吸附平衡。从样品处理时间和萃取效果两方面考虑,选择吸附时间为30 min。

**图3:**
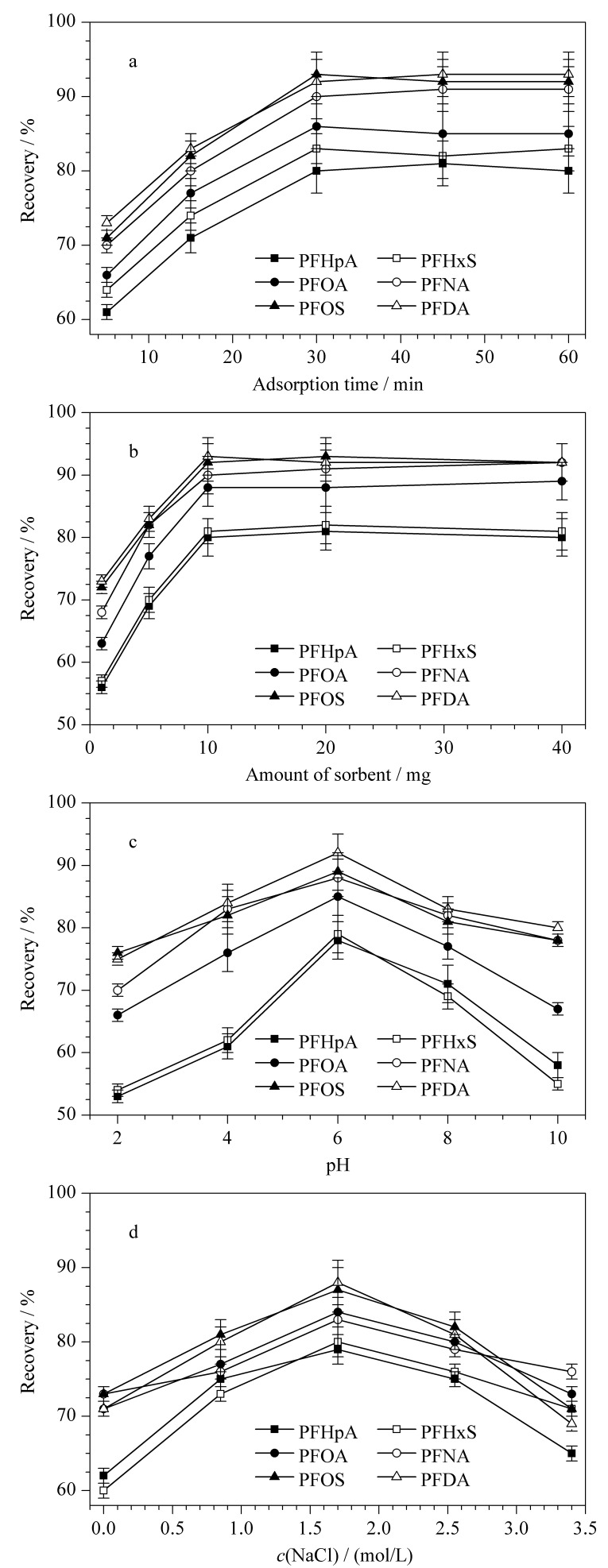
(a)吸附时间、(b)吸附剂用量、(c)样品pH值和(d)离子强度对萃取效率的影响(*n*=5)

对吸附剂用量在1~40 mg范围内进行了优化。图3b结果表明,在1~10 mg范围内,6种PFCs的回收率都随着吸附剂用量的增加而增加,在10 mg时达到最大值。在10~40 mg范围内,回收率变化不大。从绿色环保和萃取效果两方面考虑,选择吸附剂用量为10 mg。

考察了样品pH 2~10对萃取效率的影响。如图3c所示,回收率在pH为6时达到最佳水平,可能是因为PFCs在酸性和碱性溶液中羧基官能团呈质子化和去质子化状态,吸附难度增加,回收率下降。因此,选择pH为6。

调整NaCl浓度(0~3.4 mol/L),改变离子强度。如图3d所示,当NaCl浓度从1.7 mol/L增加到3.4 mol/L时,样品的回收率降低,可能由于钠离子和氯离子的竞争吸附影响了吸附剂对目标物的萃取性能。因此选择NaCl浓度为1.7 mol/L。

#### 2.3.2 解吸过程参数的优化

在解吸阶段,研究了解吸溶剂、解吸溶剂体积和解吸时间对解吸效果的影响。

考察了3种有机溶剂(甲醇、丙酮和乙腈)作为解吸溶剂的影响。如图4a所示,在这3种溶剂中,丙酮表现出对PFCs最佳的解吸效果。因此,在之后的实验中选择丙酮作为解吸溶剂。

**图4:**
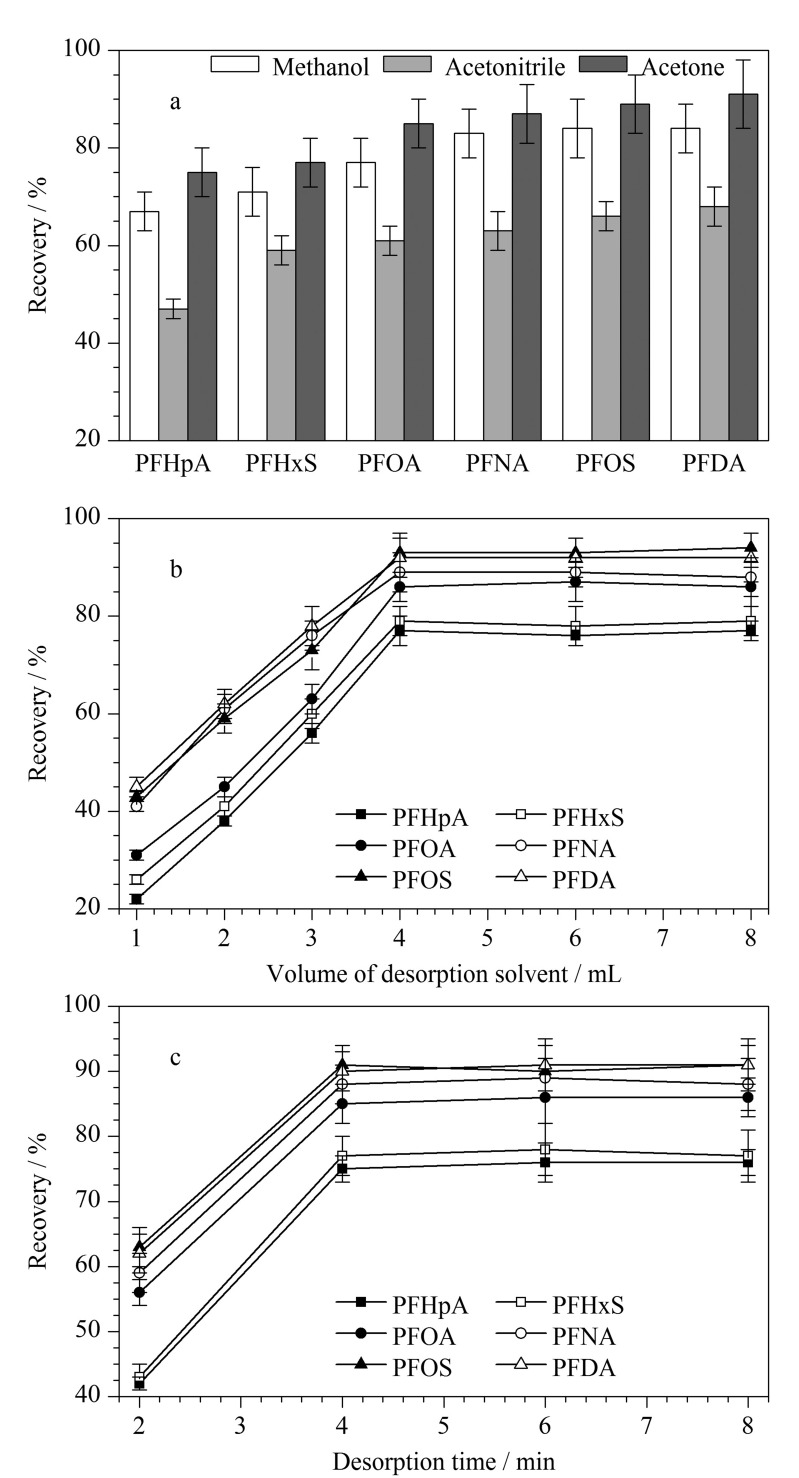
(a)解吸溶剂、(b)解吸液体积和(c)解吸时间对萃取效率的影响(*n*=5)

对丙酮体积在1~8 mL范围内进行了考察,如图4b所示,当体积为4 mL时,对PFCs的解吸效果最好。从节省溶剂和萃取效果两方面考虑,选择解吸溶剂用量为4 mL。

在2~8 min范围内考察了解吸时间,在解吸时间为4 min时,6种PFCs的回收率达到最佳(图4c)。

在最佳萃取条件下,将CNT@SiO_2_、CNT、单层吸附的(PDDA/CNT)_1_材料对PFCs的萃取结果进行了比较,CNT@SiO_2_对目标分析物的回收率最高。CNT@SiO_2_对目标分析物的回收率比CNT对目标分析物的回收率高30%~60%(图5),证明了该复合材料对目标物萃取的优越性。

**图5:**
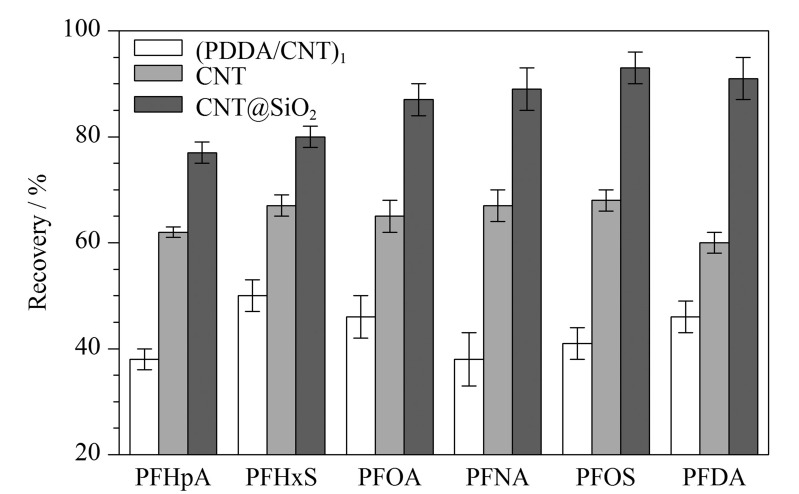
(PDDA/CNT)_1_、CNT与CNT@SiO_2_ 3种材料对PFCs萃取的回收率(*n*=3)

### 2.4 方法学参数

在最优条件下,6种PFCs在各自的线性范围内线性良好,相关系数(*r*)为0.973~0.997。方法的检出限(LOD, *S/N*=3)为0.10~0.26 ng/L,定量限(LOQ, *S/N*=10)为0.33~0.87 ng/L(表2)。

**表2:** 6种PFCs的线性范围、相关系数、检出限和定量限

Compound	Linear range/ (ng/L)	*r*	LOD/ (ng/L)	LOQ/ (ng/L)
PFHpA	0.9-1000	0.973	0.26	0.87
PFHxS	0.7-1000	0.986	0.20	0.67
PFOA	0.6-1000	0.982	0.18	0.60
PFNA	0.4-1000	0.992	0.11	0.37
PFOS	0.4-1000	0.996	0.10	0.33
PFDA	0.4-1000	0.997	0.10	0.33

在20、100和500 ng/L 3个水平下对6种PFCs进行重复性试验,日内(日间)的相对标准偏差(RSD)分别为2.73%~7.75%(3.38%~8.21%)、2.95~8.46%(4.16%~9.14%)和2.51%~7.48%(3.59%~9.63%)(*n*=3)(表3)。与其他检测水中PFCs的分析方法相比,该方法的检出限更低,线性范围更宽(表4)。对吸附材料的制备方法进行重复性试验考察,以5个不同批次制备的吸附材料作为吸附剂在相同的实验条件进行对比,目标物回收率的相对标准偏差(RSDs)为4.9%~9.3%,表明CNT@SiO_2_具有良好的制备重复性。对同一吸附材料进行可重复使用性试验,同一吸附材料连续使用8次后,目标物回收率的RSD为3.7%~8.2%,表明CNT@SiO_2_具有较好的可重复使用性。以上结果表明,CNT@SiO_2_适用于水样中6种PFCs的检测。

**表3:** 6种PFCs的日内和日间精密度(*n*=3)

Compound	20 ng/L			100 ng/L				500 ng/L						
	Intra-day RSD/%	Inter-day RSD/%		Intra-day RSD/%	Inter-day RSD/%			Intra-day RSD/%			Inter-day RSD/%			
PFHpA	7.75	8.21		6.14	8.82				5.47			7.32		
PFHxS	6.23	7.80		8.46	9.14				7.48			9.63		
PFOA	5.92	8.15		7.85	9.02				7.03			8.61		
PFNA	4.71	5.37		5.29	6.71				3.64			4.29		
PFOS	2.73	3.38		3.82	5.52				4.43			4.82		
PFDA	3.34	3.91		2.95	4.16				2.51			3.59		

**表4:** 水样中全氟化合物的分析方法比较

Adsorption materials	Extraction method	Analytical method	Linear range/ (ng/L)	LOD/ (ng/L)	Analytes	
MOFs^[1]^	d-SPE	LC-MS/MS	1-2000	0.30-2.00	PFHpA, PFHxS, PFOA, PFNA, PFOS, PFDA, PFBA,	
Bamboo charcoal^[17]^	SPE	LC-MS/MS	1-1000	0.20	PFOA	
COFs^[18]^	MSPE	LC-MS/MS	5-4000	0.62-1.39	PFHpA, PFPeA, PFOA, PFNA, PFDA, PFBA,	
EG-silicone^[19]^	SBSE	LC-MS/MS	7-177	6-50	PFHpA, PFHxA, PFOA, PFOS, PFBuA, PFPeA,	
HLB^[20]^	SPE	LC-MS/MS	500-200000	150-900	PFOS, PFOA, PFNA, PFDA	
Oasis HLB^[21]^	SPE	LC-ToF-MS	0-250	1-100	PFHpA, PFHxA, PFOA, PFNA, PFDA, PFOS, PFHxS, PFPeA	
CNT@SiO_2_(this method)	d-SPE	LC-MS/MS	0.4-1000	0.10-0.26	PFHpA, PFHxS, PFOA, PFNA, PFOS, PFDA	

MOFs: metal-organic frameworks; COFs: covalent organic frameworks; MSPE: magnetic solid-phase extraction; SBSE: stir bar sorptive extraction; EG: ethylene glycol; ToF: time-of-flight; PFBA: perfluorobutyric acid; PFPeA: perfluoropentanoic acid; PFHxA: perfluorohexanoic acid; PFBuA: perfluorobutanoic acid.

### 2.5 实际水样中PFCs的分析

为了评价本方法的适用性,选择3种实际环境水样(自来水、桶装饮用水和河水),采用优化后的方法进行检测分析。所有水样经过0.22 μm的水相微孔滤膜过滤后,存储于棕色的干净玻璃瓶中,并放置于4 ℃下保存。结果表明,自来水中检测出PFOA和PFOS的含量分别为5.6 ng/L和8.7 ng/L;在桶装饮用水和河水中没有检测到6种PFCs。在3种空白环境水样中添加3个水平的PFCs(10、100和500 ng/L),回收率为72.1%~109.6%(表5)。图6为自来水加标样品的色谱图。

**表5:** 环境水样中6种PFCs的加标回收率(*n*=5)

Compound	Added level/ (ng/L)	Recoveries/%			Compound	Added level/ (ng/L)	Recoveries/%		
		Tap water	Barreled drinking water	River water			Tap water	Barreled drinking water	River water
PFHpA	10	72.4±2.3	73.4±5.6	77.1±9.0	PFNA	10	85.4±3.3	82.8±7.5	85.1±2.9
	100	72.1±2.5	74.1±5.0	72.9±8.0		100	84.1±3.1	85.5±3.1	83.8±3.7
	500	74.2±4.1	77.0±8.0	80.3±7.9		500	82.1±1.9	85.2±6.9	91.9±7.3
PFHxS	10	75.1±6.2	77.5±7.3	81.5±8.0	PFOS	10	87.2±5.9	93.1±6.1	103.0±8.5
	100	76.6±8.5	81.1±4.4	79.3±3.8		100	93.5±5.1	100.8±9.6	90.6±8.0
	500	80.6±2.9	79.6±6.9	76.7±2.9		500	86.8±4.5	92.9±8.1	89.6±8.0
PFOA	10	83.6±6.1	76.1±6.2	81.0±4.1	PFDA	10	89.6±8.5	88.0±3.9	90.3±3.9
	100	81.4±6.2	83.2±8.1	82.0±7.8		100	86.6±9.0	92.8±3.0	86.4±3.5
	500	86.2±9.1	82.7±3.7	84.6±8.0		500	109.6±7.1	84.9±6.4	94.8±2.8

**图6:**
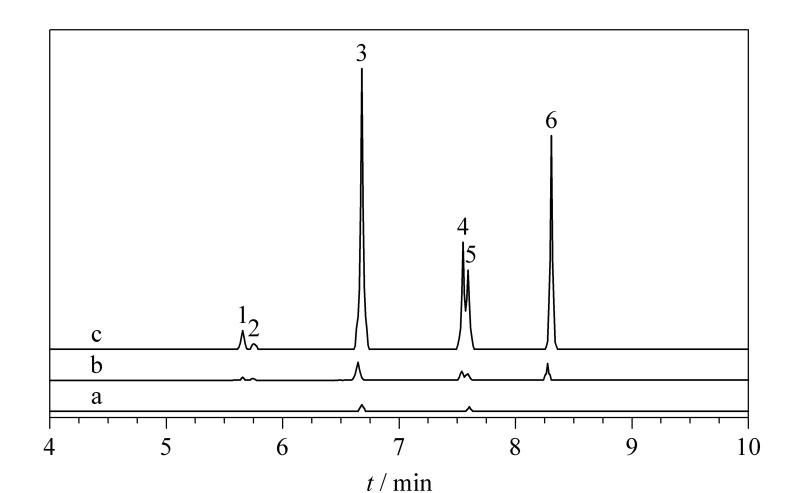
(a)空白自来水及其加标(b)10 ng/L和(c)500 ng/L PFCs后的色谱图

## 3 结论

本文采用一种新型的CNT@SiO_2_材料作为吸附剂,基于d-SPE前处理技术和LC-MS/MS,建立了一种高效、灵敏的分析环境水样中痕量全氟化合物的新方法。与现有的PFCs检测方法相比,本方法检出限低,线性范围宽,萃取效率高,重复性好。本方法应用于环境水样中痕量全氟化合物的分析,取得了满意的结果。
